# Psychological assessments, allostatic load and gene expression analyses in a randomized controlled trial comparing meditation, yoga, and stress education

**DOI:** 10.3389/fpsyg.2025.1653242

**Published:** 2025-09-12

**Authors:** John W. Denninger, Diane Joss, Perla M. Romero, Sat Bir Singh Khalsa, Elizabeth A. Hoge, Manoj Bhasin, Sara W. Lazar, Jeffery A. Dusek, Eric Macklin, Towia Libermann, Gregory L. Fricchione, Herbert Benson

**Affiliations:** ^1^Benson-Henry Institute for Mind Body Medicine, Massachusetts General Hospital, Boston, MA, United States; ^2^Harvard Medical School, Boston, MA, United States; ^3^Department of Psychiatry, Massachusetts General Hospital, Boston, MA, United States; ^4^Department of Medicine, Brigham and Women’s Hospital, Harvard Medical School, Boston, MA, United States; ^5^Department of Psychiatry, Georgetown University Medical Center, Washington, DC, United States; ^6^Department of Biomedical Informatics, Emory University School of Medicine, Atlanta, GA, United States; ^7^Susan Samueli Integrative Health Institute, University of California-Irvine, Irvine, CA, United States; ^8^School of Medicine, University of California-Irvine, Irvine, CA, United States; ^9^Genomics, Proteomics, Bioinformatics and Systems Biology Center, Beth Israel Deaconess Medical Center, Boston, MA, United States

**Keywords:** biomarker, contemplative, allostatic load, inflammation, gene, rct

## Abstract

**Objectives:**

The mind–body research field has explored a broad range of outcome measures, however, there has not been systematic investigation on these outcome measures and there is little knowledge on what outcome measures can capture the differences between different mind–body practices. Therefore, this three-arm randomized controlled trial examined the effects of meditation vs. yoga vs. an active control condition of stress education, with a large battery of outcome measures including psychosocial self-report variables, allostatic load biomarkers, and gene expression measures.

**Methods:**

A total of 211 chronically stressed but otherwise healthy adults were randomized to 8-week one-on-one in-person interventions of meditation (*N* = 73), yoga (*N* = 68) or stress education (*N* = 70) interventions. Between-group differences in psychological outcome measurements, allostatic load biomarkers and genomic measures were compared at baseline (week 0), post-intervention (week 9), and at 26-week follow-up. Data were analyzed using a shared-baseline, two-way, repeated-measures ANOVA with unstructured within-person covariance over measurement timepoints. Treatment and time-specific effects were estimated using linear contrasts of adjusted means. False discovery rate correction was applied for multiple comparisons.

**Results:**

None of the outcome measures had significant differences among the three treatment arms. Within each treatment arm, most psychological questionnaire measures showed significant improvements (corrected *p* < 0.05). IL-6 showed slight elevation (but still within normal range for healthy adults) at the post-intervention timepoint within the stress education arm (corrected *p* < 0.05) and at the follow-up timepoint within the meditation arm (corrected *p* < 0.05). High density lipoprotein (HDL) levels were increased within the yoga arm at the follow-up timepoint (corrected *p* < 0.05). Post-intervention score changes of the Perceived Stress Scale (PSS) correlated with blood pressure changes in the meditation arm, insulin level changes in the yoga arm, and changes of allostatic load index in the control arm, none of which survived correction for multiple comparisons.

**Conclusion:**

This study did not find any significant between-group effects with any outcome measures. The null findings in this study might have been due to floor effects from the study sample of healthy adults.

**Clinical trial registration:**

https://clinicalTrials.gov, identifier: NCT01308970.

## Introduction

Mind–body practices have been shown to have therapeutic effects in a wide variety of physical and psychological conditions, such as stress, depression, and anxiety (D'Silva et al., 2012; Maric et al., 2021). Despite growing research interest in the psychophysiology of mind–body practices (Brandmeyer et al., 2019; Riley and Park, 2015), there has been little consensus on the choice of mechanistic or therapeutic outcome variables, and there are rarely direct comparisons on the effects of different mind–body modalities (e.g., meditation vs. yoga) that are popular among US adults (Dossett et al., 2020). Therefore, we conducted a randomized controlled trial (RCT) to comprehensively compare meditation and yoga against an active control condition of stress education, with a large battery of outcome measures including self-report psychological questionnaires, stress biomarkers and gene expression measures.

A large number of self-report questionnaires have been used in mind–body research (Crosswell and Lockwood, 2020). The Perceived Stress Scale (PSS) is one of the most widely used self-report measures of stress (Cohen et al., 1983). Additional measures on depression (Zigmond and Snaith, 1983), anxiety (Spielberger et al., 1971), burnout and resilience (Wagnild and Young, 1993) are also frequently utilized. Therefore, this study included a large battery of psychological measures to comprehensively evaluate their reliability in detecting psychological symptom changes across different mind–body modalities.

“Allostatic load” is a concept regarding the accumulative “wear and tear” of chronic stress on physiological systems (McEwen and Stellar, 1993; Prior, 2021). While the human body can adapt to mild levels of stress, repeated and prolonged environmental challenges can eventually lead to inability to maintain a normal homeostatic balance due to chronic alterations in physiological systems (McEwen and Stellar, 1993; Prior, 2021). Long-term adverse effects of maladaptive response to chronic stress (Dhabhar, 2014) include inflammation and immunosuppression (Barrett et al., 2021), cardiovascular dysfunction and disease (Kivimäki and Steptoe, 2018), accumulation of abdominal fat (Aschbacher et al., 2014), loss of bone minerals (Kelly et al., 2019), decreased neurogenesis (Du Preez et al., 2021), increased neuronal cell death and associated atrophy in the limbic system (Schoenfeld et al., 2017). Many stress biomarkers have been shown to respond to mind–body practices, for example, prior mind–body studies have shown changes with interleukin-6 (IL-6) levels, despite having mixed findings (Bower and Irwin, 2016; Moreno, 2024). Cortisol is another biomarker that is frequently investigated in mind–body research which also has inconclusive findings in the literature (O'Leary et al., 2016). Mindfulness-based interventions have been shown to reduce blood pressure (Intarakamhang et al., 2020; Solano López, 2018). Mind–body practices have also shown promises in weight management (López-Alarcón et al., 2025). Due to the multi-facet effects of mind–body practices and the potential limitation with a particular biomarker, in this study we utilized a composite “allostatic load index” to capture biomarkers from several physiological systems including the neuroendocrine system, the immune system, the cardiovascular system, and the metabolic system (Logan and Barksdale, 2008; Seeman et al., 2010). Using such a composite measure to reflect stress-related dysregulation across several physiological systems increases measure reliability.

Inflammatory genetic transcription factors are also well-researched biomarkers of chronic stress (Natoli et al., 2011). These transcription factors regulate inflammatory and anti-inflammatory gene expression through modulating the production of cytokines, chemokines, and other inflammatory molecules (Cohen et al., 2012). Notable transcription factors include nuclear factor kappa-light-chain-enhancer of activated B cells (NF-κB) (Biancalana et al., 2021) anti-inflammatory and pro-inflammatory pathways, as well as tumor necrosis factor receptor 2 (TNFR2; Medler and Wajant, 2019), and IL-6 JAK STAT3 signaling (Johnson et al., 2018) that are indicative of inflammatory and immune responses. Prior mind–body research demonstrated reduction of the NF-κB pro-inflammatory transcription control pathway (Bhasin et al., 2013; Dutcher et al., 2022; Oyler et al., 2023) as well as the TNFR2 pathway (Bhasin et al., 2013). Therefore, this study also measured several genomic biomarkers from blood samples and evaluated their relationships with self-report measures.

This study utilized a three-arm RCT design to compare the effects of meditation vs. yoga vs. an active control of stress education, to identify potential common and unique psychophysiological effects of meditation and yoga. The study was conducted with chronically stressed but otherwise healthy adults to focus on stress-related effects and mechanisms while minimizing the influences from pre-existing disease conditions.

## Materials and methods

### Overview of study procedures

**Figure 1:**
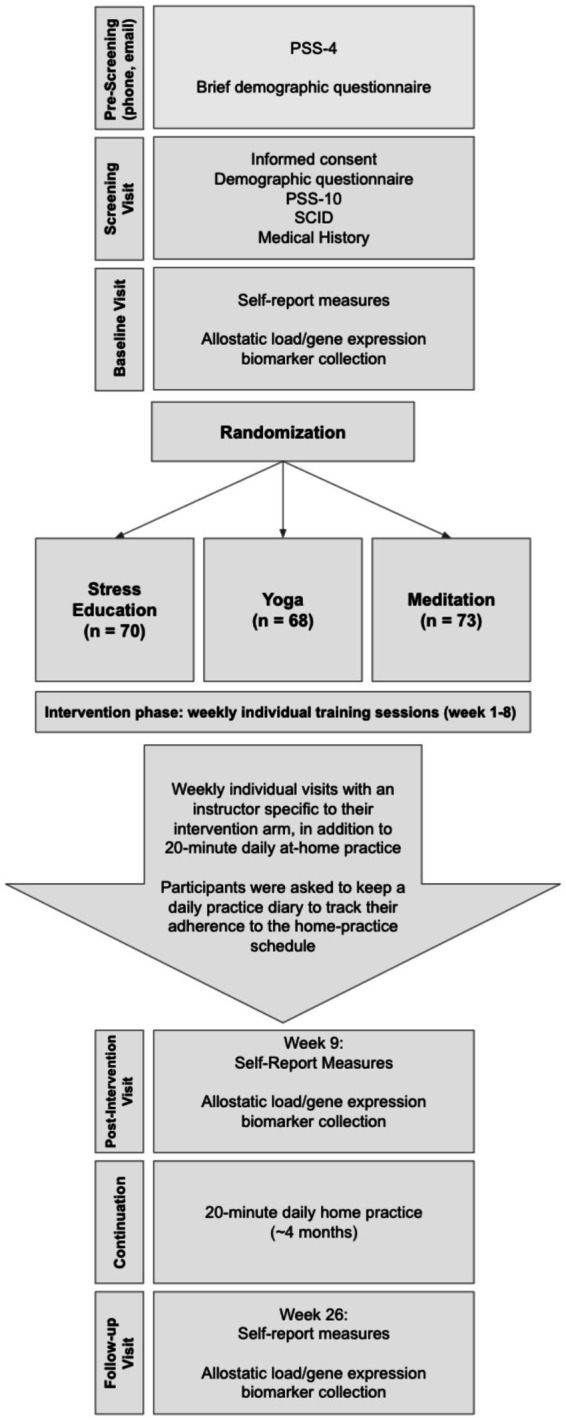
Diagram of study procedures.

This study was conducted according to a research grant awarded by the National Institute of Health (see Supplementary material). The clinical trial was registered on ClinicalTrials.gov (NCT01308970). All study procedures were approved by the Institutional Review Board (IRB) of Mass General Brigham. All participants provided written informed consent according to IRB-approved consenting procedure with the IRB-approved consent form. The overall study procedures included participant pre-screening with the 4-item version of the Perceived Stress Scale (PSS-4; PSS-4 score ≥5 required to pass pre-screening), consenting, enrollment, and randomization into three arms: meditation, yoga, or stress education as an active control condition, each of which consisted of 8 weeks of one-on-one in person training. Psychological measures and biological samples/measures were collected at the baseline visit (week 0), post-intervention visit (week 9) and follow-up visit (week 26; Figure 1).

Eligibility criteria included: age 18 years or above, PSS-10 score ≥16 for males and ≥17 for females, and self-reporting of chronic stress for ≥6 months. Candidate participants were excluded for any major medical disorder, abnormality, or condition that would preclude a safe and effective yoga and meditation practice, any psychopathology as determined from structured clinical interview for DSM-IV-TR Axis I disorders (SCID; First et al., 2005), cognitive impairment, pregnancy, and the use of psychoactive medications (with the exception of hypnotics) and medications that might interfere with genetic expression (e.g., steroids, NSAIDs if used >3 days per week, and immunosuppressive or cytotoxic therapies such as chemotherapy for cancer). Patients were also excluded if they were already engaged in regular practice of mind–body techniques.

### Study interventions

Participants in each arm received weekly one-on-one in-person training for 8 weeks with a study interventionist. Each weekly session was 1-h long, and every session had the same structure depending on the intervention arm. All participants were also instructed to complete a daily 20-min home practice. After the end of the 8-week interventions, participants were required to continue their assigned 20-min daily home practice for ~4 months until completion of their follow-up study visit.

#### Meditation

The meditation intervention was based upon concentrative meditation practice as has been applied in the “relaxation response” technique developed and routinely utilized at the Benson-Henry Institute for Mind Body Medicine (Benson et al., 1974). The practice involved using a mental focal point coordinated with the breath to help focus and shift attention back from mind wandering while applying a quiet, aware, but non-judging, non-analytical mental attitude. The focal point can be a short word (e.g., one, peace, calm, love), phrase (e.g., in-out, rising-falling), prayer, sound, or the repetitive rhythm of the breath.

Every session started with 10 min of stress education, followed by 45 min for instructions and meditative practices. Instruction topics included the theory, science, and benefits of meditation and different types of meditation. All sessions concluded with 5 min of questions and answers. Participants were supplied with audio recordings for the daily 20-min home practice of concentration meditation.

#### Yoga

The yoga intervention used Kundalini Yoga (as taught by Yogi Bhajan), a widely utilized school of yoga practice that emphasizes meditation and breathing practices (Shannahoff-Khalsa, 2004), with typical practices including physical postures and movement exercises, breath regulation techniques, deep relaxation, and meditation and mindfulness practices including mantra and chanting. Weekly sessions started with 10 min of stress education followed by 45 min of practices that included a specific yoga set (~30 min) followed by a breathing meditation (~8 min) and a deep supine relaxation (corpse pose, ~7 min). Participants were given audio recordings for the daily 20-min home practice that included 9 min of simple physical postures/exercises emphasizing flexibility and tension release followed by an 11-min slow breathing meditation in a specific seated posture with an alternating pattern of slow breathing through the mouth and nose.

#### Stress education as attention control

The stress education control condition was successfully utilized in prior mind–body research (Dusek et al., 2008a; Dusek et al., 2008b; Hoge et al., 2013). Participants were provided with detailed and extensive information about stress and health. Information on yoga or meditation-based stress education techniques (e.g., meditation, breathing, body awareness or imagery) was not provided. The educational content included definitions of stress and the stress response, the fight or flight response, physiological and psychological effects of stress, stress and performance, the negative stress cycle, stress and health/illness, stress and immunity, stress buffers, stress hardiness, stress and heart disease, the role of genes and environment in health, the contribution of lifestyle behaviors such as caffeine and alcohol intake and cigarette smoking, and the importance of regular exercise and proper diet. To control for time and attention spent in the meditation and yoga interventions on daily home practice, subjects were asked to sit quietly in a comfortable position and listen for 20 min daily to audiobooks related to stress management.

### Outcome measures

The goal of this study was to investigate the characteristics of commonly used outcome measures in stress reduction research and their relationships, including a comprehensive battery of (1) self-report psychological measures, (2) biochemical assays quantifying biomarkers from blood and urine samples for assessing allostatic load, and (3) gene expression measures from blood samples.

#### Self-report psychological questionnaires

PSS (Cohen et al., 1983): PSS is a well-validated and widely used survey that evaluates the degree to which a subject appraises recent experiences as stressful. The 4-item version (PSS-4) was utilized for pre-screening, and the 10-item version (PSS-10) was used as a primary outcome measure.

Symptom Checklist 90-Revised (SCL-90R; Derogatis and Savitz, 1999): SCL-90R is a 90-item inventory for assessing the following psychopathological symptom dimensions: somatization, obsessive-compulsive, interpersonal sensitivity, depression, anxiety, hostility, phobic anxiety, paranoid ideation, psychoticism and a category of additional dimensions. Three global indices can be obtained. The primary global index is the Global Severity Index (GSI) which is a weighted frequency score calculated by averaging the scores of the 9 dimensions over the total number of answered items. The Positive Symptom Total (PST) is the total number of items with non-zero responses and the Positive Symptom Distress Index (PSDI) is the sum of the non-zero scores divided by the PST.

The Psychological Well-Being Scales (PWBS; Ryff, 1989): PWBS is a 84-item instrument that measures different facets of psychological well-being such as autonomy (AUTO), environmental mastery (EM), personal growth (PG), positive relations with others (PR), purpose in life (PL), and self-acceptance (SA).

Wagnild and Young Resilience Scale (W&Y; Wagnild and Young, 1993): This 26-item questionnaire assesses five components of resiliency: equanimity reflecting the ability to adapt to difficult circumstances, perseverance, self-reliance, meaningfulness, and existential loneliness or sense of uniqueness.

The Positive and Negative Affect Scales (PANAS; Watson et al., 1988): PANAS includes two 10-item scales for measuring positive (PA) and negative affect (NA).

State–Trait Anxiety Inventory-State Score (STAI-SS; Spielberger et al., 1971): STAI-SS is a concise 20-item questionnaire for measuring situational or state anxiety.

Hospital Anxiety and Depression Scale (HADS; Zigmond and Snaith, 1983): HADS is a 14-item questionnaire for assessing anxiety (HADS-A) and depression (HADS-D) in the setting of a medical practice.

Cognitive and Affective Mindfulness Scale-Revised (CAMS-R; Feldman et al., 2007): CAMS-R is a 12-item scale that measures everyday mindfulness, with subscales on attention (Att), present-focus (PF), awareness (Awr) and acceptance (Acc).

#### Allostatic load index

The 14 individual components of the allostatic load index (Seeman et al., 2010) consist of measures of the HPA axis (cortisol, DHEA-S), the sympatho-adrenal-medullary pathway (epinephrine and norepinephrine), chronic stress (blood pressure), inflammation (C-reactive protein and interleukin-6), cardiovascular health [resting pulse, cholesterol, high density lipoprotein (HDL)], and metabolism [insulin resistance, hemoglobin A1c (HbA1c), body mass index (BMI), waist-hip ratio]. During each of the three study visits, which all took place on weekday mornings, a study nurse collected fasting blood samples and measured blood pressure, waist-hip ratio, resting pulse, and height and weight.

The total amount of urinary cortisol, epinephrine and norepinephrine secreted over 12-h periods were collected on 3 consecutive normal weekdays prior to each study visit. Participants were instructed to collect all urine within the 12 h before their habitual wake time. The total excreted cortisol, epinephrine, and norepinephrine was calculated by determining the urine concentration of all three hormones in this urine sample.

On the morning that urinary samples were brought in at each study visit, participants had fasting blood draw for the measurement of C-reactive protein, interleukin-6, interleukin-1b, DHEA-S, insulin, HbA1c, cholesterol panel, oxytocin and TNF-alpha. Blood was collected by venipuncture into tubes containing clot activator and tubes containing EDTA and placed on ice. Clot activator tubes were allowed to clot for 30 min. Tubes were then centrifuged at 1500 rpm for 10 min at 4°C. Serum and plasma were transferred into 2 cc plastic tubes, capped and frozen at −70°C until processing. The cholesterol panel, insulin, HbA1c, and C-reactive protein were assayed using paramagnetic particle chemiluminescent immunoassay, radioimmunoassay or enzyme-linked immunosorbent assay (ELISA) kits. Levels of IL-6, DHEA-S, and TNF-alpha were also assayed using commercially available chemiluminescent immunoassay, radioimmunoassay or ELISA kits.

#### Genomics

Additional blood samples were collected at each study visit for whole blood transcriptional profiling. Blood was drawn directly into 2 separate 2.5 mL PaxGene Blood RNA tubes (Qiagen, Stanford, CA), incubated at room temperature for at least 2 h and processed for total RNA isolation according to the manufacturer’s protocol. Samples were processed at the Genomics Center at Beth-Israel Deaconess Medical Center. RNA purification, cDNA synthesis and *in vitro* transcription for production of biotin-labeled cRNA was also performed using the CodeLink Expression Assay Reagent kit (GE Healthcare).

### Statistical analysis

Data were analyzed using a shared-baseline, two-way repeated-measures ANOVA with unstructured within-person covariance over visits. The model adjusted for participant sex, baseline age and PSS-10. Treatment and time-specific effects were estimated using linear contrasts of adjusted means. All randomized participants were included, classified according to the treatment they received, and analyzed by maximum likelihood. False discovery rate correction was applied for multiple comparisons.

## Results

### Participants characteristics at baseline

**Figure 2:**
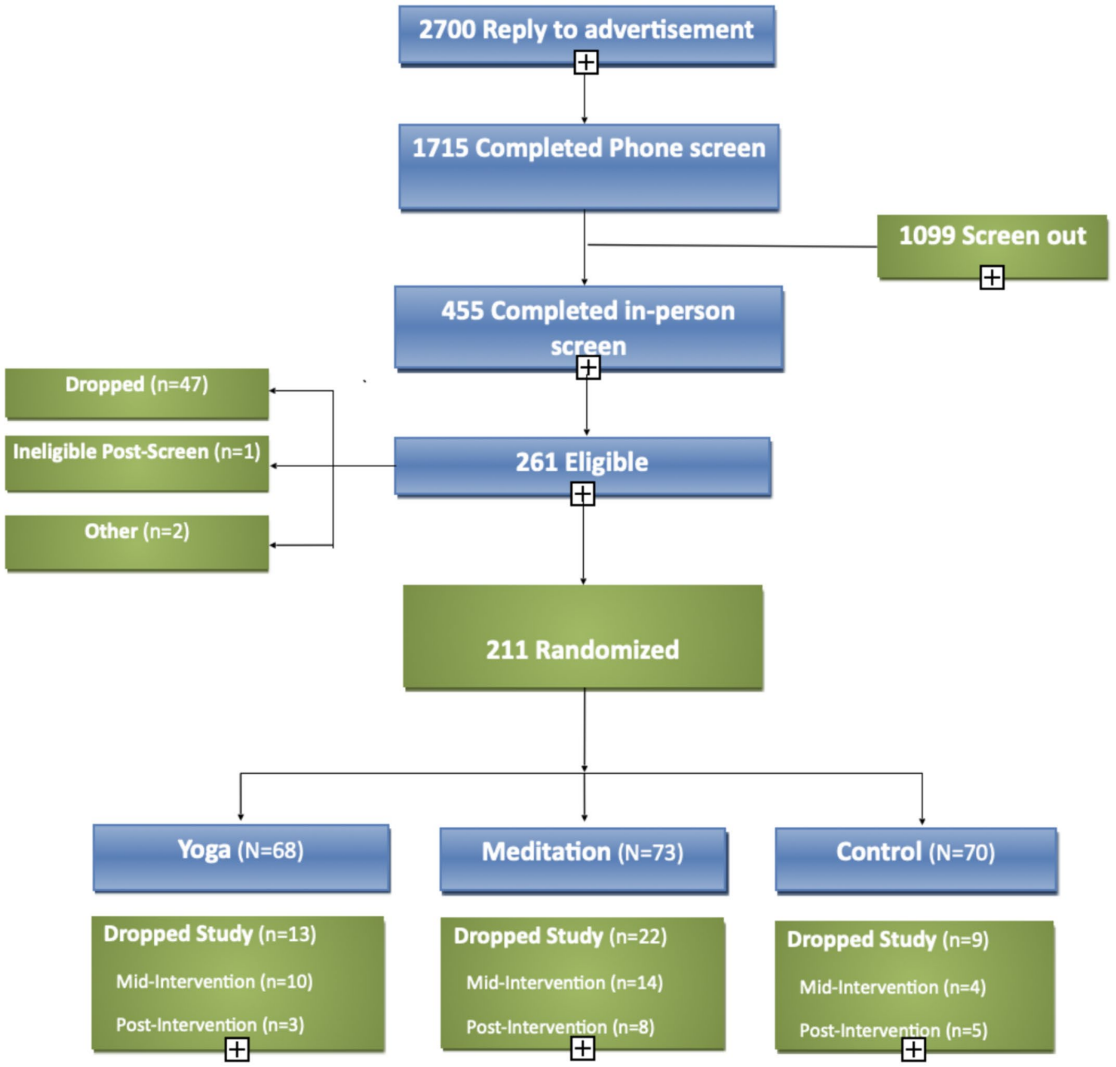
Consort diagram of research participant screening, enrollment and retention.

A total of 211 healthy adults with chronic stress were enrolled and randomized (Figure 2). The mean age was 36 ± 14 years, and the majority of participants were white (73%), non-Hispanic (89%), female (63%; Table 1). There were no significant differences in baseline scores on any psychological measures across the three treatment arms.

**Table 1:** Demographic characteristics of the sample in each intervention arm.

Characteristic	Overall (*n* = 211)	Stress education (*n* = 70)	Meditation (*n* = 73)	Yoga (*n* = 68)
	N (%)	N (%)	N (%)	N (%)
Gender				
Female	133 (63.0)	43 (61.4)	46 (63.0)	44 (64.7)
Male	78 (37.0)	27 (38.6)	27 (37.0)	24 (35.3)
Race				
White	152 (72.0)	54 (77.1)	51 (69.9)	47 (69.2)
Asian	23 (10.9)	7 (10.0)	7 (9.6)	9 (13.2)
Black or African American	18 (8.5)	4 (5.7)	7 (9.6)	7 (10.3)
American Indian or Alaska Native	2 (1.0)	0 (0.0)	2 (2.7)	0 (0.0)
Multiracial	13 (6.2)	4 (5.7)	6 (8.2)	3 (4.4)
Missing	3 (1.4)	1 (1.5)	0 (0.0)	2 (2.9)
Ethnicity				
Hispanic or Latino	23 (10.9)	10 (14.3)	3 (4.1)	10 (14.7)
Not Hispanic or Latino	188 (89.1)	60 (85.7)	70 (95.9)	58 (85.3)
	M(SD)	M(SD)	M(SD)	M(SD)
Age at Baseline	36 (14.1)	36.5 (14.0)	36.4 (15.0)	35.1 (13.2)

### Psychological outcomes

As the primary psychometric outcome measure, PSS-10 showed significant improvement in each of the three arms at post-intervention and follow-up timepoints (raw *p* < 0.001, Table 2, corrected *p* < 0.05) without significant between-group effects. Most of other psychological outcome measures also showed significant improvement within each arm (raw *p* < 0.001, Table 2, corrected *p* < 0.05), but without significant between-group effects except for PWBS-PR which had greater improvement in yoga compared to control (raw *p* = 0.005), which did not survive correction for multiple comparisons.

**Table 2:** Change in psychosocial measures scores from baseline (week 0) to immediate end of treatment (week 9), and from baseline (week 0) to follow-up (week 26).

	Baseline			Post-intervention vs. baseline			Follow-up vs. baseline		
	Est	SE	*p*	Est	SE	*p*	Est	SE	*p*
Overall									
PSS-10	21.64	0.33	-	−5.18	0.54	<0.001	−5.87	0.55	<0.001
SCL-90R GSI	0.55	0.02	-	−0.16	0.02	<0.001	−0.18	0.03	<0.001
SCL-90R PST	30.4	1.01	-	−6.75	1.02	<0.001	−7.96	1.14	<0.001
SCL-90R PSDI	1.59	0.02	-	−0.13	0.02	<0.001	−0.11	0.03	0.001
PWBS–Auto	61.03	0.76	-	1.66	0.55	0.003	1.81	0.65	0.005
PWBS–EM	53.44	0.69	-	5.29	0.68	<0.001	7.70	0.80	<0.001
PWBS–PG	70.19	0.57	-	2.13	0.45	<0.001	2.35	0.53	<0.001
PWBS–PR	64.09	0.78	-	1.51	0.59	0.012	2.29	0.68	<0.001
PWBS–PL	64.97	0.73	-	2.41	0.55	<0.001	3.95	0.67	<0.001
PWBS–SA	57.28	0.84	-	4.18	0.67	<0.001	5.71	0.77	<0.001
PANAS–PA	33.65	0.46	-	1.91	0.46	<0.001	2.57	0.52	<0.001
PANAS–NA	21.44	0.38	-	−2.58	0.44	<0.001	−3.30	0.46	<0.001
W&Y	129.0	1.45	-	4.85	2.05	0.019	3.29	2.58	0.204
STAI-SS	42.01	0.70	-	−4.82	0.85	<0.001	−6.25	0.95	<0.001
HADS-A	8.04	0.23	-	−1.81	0.28	<0.001	−2.14	0.30	<0.001
HADS-D	5.03	0.21	-	−1.10	0.24	<0.001	−1.46	0.25	<0.001
CAMS-R Att	7.75	0.15	-	0.50	0.17	<0.001	0.77	0.17	<0.001
CAMS-R PF	8.15	0.12	-	0.48	0.12	<0.001	0.75	0.13	<0.001
CAMS-R Awr	7.49	0.14	-	0.67	0.15	<0.001	0.78	0.16	<0.001
CAMS-R Acc	7.87	0.14	-	0.56	0.15	<0.001	0.70	0.17	<0.001
CAMS-R Tot	31.24	0.41	-	2.28	0.66	<0.001	3.05	0.47	<0.001
Stress education									
PSS-10	21.64	0.33	-	−5.20	0.78	<0.001	−6.04	0.81	<0.001
SCL-90R GSI	0.55	0.02	-	−0.15	0.04	<0.001	−0.16	0.04	<0.001
SCL-90R PST	30.4	1.01	-	−5.54	1.62	<0.001	−7.01	1.77	<0.001
SCL-90R PSDI	1.59	0.02	-	−0.10	0.04	0.007	−0.08	0.05	0.101
PWBS–Auto	61.03	0.76	-	1.08	0.88	0.22	2.46	1.01	0.016
PWBS–EM	53.44	0.69	-	4.72	1.08	<0.001	7.86	1.25	<0.001
PWBS–PG	70.19	0.57	-	2.18	0.69	0.002	2.68	0.80	0.001
PWBS–PR	64.09	0.78	-	−0.58	0.97	0.55	0.95	1.08	0.378
PWBS–PL	64.97	0.73	-	1.70	0.86	0.049	3.44	1.03	<0.001
PWBS–SA	57.28	0.84	-	3.41	1.09	0.002	5.38	1.19	<0.001
PANAS–PA	33.65	0.46	-	1.36	0.74	0.068	2.84	0.83	<0.001
PANAS–NA	21.44	0.38	-	−3.24	0.70	<0.001	−3.77	0.70	<0.001
W&Y	129.0	1.45	-	4.68	3.09	0.132	5.22	3.99	0.192
STAI-SS	42.01	0.70	-	−4.92	1.31	<0.001	−6.16	1.47	<0.001
HADS-A	8.04	0.23	-	−2.34	0.43	<0.001	−2.35	0.46	<0.001
HADS-D	5.03	0.21	-	−1.12	0.37	0.003	−1.55	0.38	<0.001
CAMS-R Att	7.75	0.15	-	0.30	0.23	0.197	0.52	0.26	0.047
CAMS-R PF	8.15	0.12	-	0.75	0.18	<0.001	0.66	0.20	0.001
CAMS-R Awr	7.49	0.14	-	0.85	0.23	<0.001	0.77	0.25	0.002
CAMS-R Acc	7.87	0.14	-	0.48	0.23	0.04	0.43	0.27	0.11
CAMS-R Tot	31.24	0.41	-	2.28	0.67	<0.001	2.36	0.73	0.002
Meditation									
PSS-10	21.64	0.33	-	−4.40	0.80	<0.001	−4.48	0.86	<0.001
SCL-90R GSI	0.55	0.02	-	−0.15	0.04	<0.001	−0.19	0.04	<0.001
SCL-90R PST	30.4	1.01	-	−6.19	1.73	<0.001	−8.28	1.95	<0.001
SCL-90R PSDI	1.59	0.02	-	−0.15	0.04	<0.001	−0.11	0.06	0.06
PWBS–Auto	61.03	0.76	-	1.59	0.93	0.089	0.56	1.11	0.615
PWBS–EM	53.44	0.69	-	5.88	1.15	<0.001	6.77	1.37	<0.001
PWBS–PG	70.19	0.57	-	1.69	0.73	0.022	1.70	0.89	0.056
PWBS–PR	64.09	0.78	-	1.66	1.03	0.107	1.70	1.18	0.152
PWBS–PL	64.97	0.73	-	2.57	0.91	0.005	3.19	1.13	0.005
PWBS–SA	57.28	0.84	-	4.54	1.15	<0.001	5.25	1.31	<0.001
PANAS–PA	33.65	0.46	-	1.89	0.78	0.016	1.79	0.92	0.054
PANAS–NA	21.44	0.38	-	−2.02	0.74	0.007	−2.86	0.77	<0.001
W&Y	129.0	1.45	-	4.65	3.27	0.157	2.58	4.44	0.562
STAI-SS	42.01	0.70	-	−4.40	1.39	0.002	−5.42	1.63	0.001
HADS-A	8.04	0.23	-	−1.41	0.46	0.002	−1.19	0.50	0.019
HADS-D	5.03	0.21	-	−0.61	0.39	0.123	−0.74	0.41	0.073
CAMS-R Att	7.75	0.15	-	0.47	0.24	0.057	0.73	0.28	0.01
CAMS-R PF	8.15	0.12	-	0.30	0.19	0.122	0.34	0.22	0.12
CAMS-R Awr	7.49	0.14	-	0.37	0.24	0.13	0.46	0.28	0.096
CAMS-R Acc	7.87	0.14	-	0.36	0.24	0.14	0.42	0.29	0.155
CAMS-R Tot	31.24	0.41	-	1.62	0.70	0.021	2.09	0.80	0.01
Yoga									
PSS-10	21.64	0.33	-	−5.95	0.84	<0.001	−7.09	0.86	<0.001
SCL-90R GSI	0.55	0.02	-	−0.19	0.04	<0.001	−0.20	0.04	<0.001
SCL-90R PST	30.4	1.01	-	−8.52	1.75	<0.001	−8.61	1.89	<0.001
SCL-90R PSDI	1.59	0.02	-	−0.15	0.04	<0.001	−0.13	0.06	0.02
PWBS–Auto	61.03	0.76	-	2.30	0.95	0.016	2.42	1.09	0.027
PWBS–EM	53.44	0.69	-	5.26	1.16	<0.001	8.48	1.34	<0.001
PWBS–PG	70.19	0.57	-	2.51	0.74	<0.001	2.67	0.86	0.002
PWBS–PR	64.09	0.78	-	3.44	1.04	0.001	4.21	1.16	<0.001
PWBS–PL	64.97	0.73	-	2.96	0.93	0.002	5.23	1.10	<0.001
PWBS–SA	57.28	0.84	-	4.59	1.17	<0.001	6.50	1.28	<0.001
PANAS–PA	33.65	0.46	-	2.48	0.79	0.002	3.09	0.89	<0.001
PANAS–NA	21.44	0.38	-	−2.47	0.75	0.001	−3.27	0.75	<0.001
W&Y	129.0	1.45	-	5.23	3.32	0.117	2.06	4.28	0.631
STAI-SS	42.01	0.70	-	−5.13	1.41	<0.001	−7.17	1.57	<0.001
HADS-A	8.04	0.23	-	−1.67	0.46	<0.001	−2.88	0.49	<0.001
HADS-D	5.03	0.21	-	−1.58	0.40	<0.001	−2.08	0.41	<0.001
CAMS-R Att	7.75	0.15	-	0.73	0.25	0.004	1.07	0.28	<0.001
CAMS-R PF	8.15	0.12	-	0.39	0.20	0.051	1.24	0.21	<0.001
CAMS-R Awr	7.49	0.14	-	0.73	0.25	0.004	1.10	0.27	<0.001
CAMS-R Acc	7.87	0.14	-	0.84	0.25	<0.001	1.26	0.28	<0.001
CAMS-R Tot	31.24	0.41	-	2.62	0.71	<0.001	4.69	0.78	<0.001

Uncorrected raw *p*-values are reported in the table, those with *p* ≤ 0.001 survived correction for multiple comparisons with corrected *p* < 0.05. Est, estimated means (i.e., estimates at baseline are the same across group interventions given that this is a shared-baseline analysis); SE, standard error; PSS, Perceived Stress Scale; SCL-90R, Symptom Checklist 90-Revised; GSI, The Global Severity Index; PST, The Positive Symptom Total Index; PSDI, The Positive Symptom Distress Index; PWBS, The Psychological Well-Being Scales; Auto, autonomy; EM, environmental mastery; PG, personal growth; PR, positive relations; PL, purpose in life; SA, self-acceptance; PANAS, The Positive and Negative Affect Scales; PA, positive affect; NA, negative affect; W&Y, Wagnild and Young Resiliency Scale; STAI-SS, State–Trait Anxiety Inventory Scale-State Score; HADS-A, Hospital Anxiety and Depression Scale-Anxiety Subscale; HADS-D, Hospital Anxiety and Depression Scale-Depression Subscale; CAMS-R, Cognitive and Affective Mindfulness Scale-Revised; Att, attention; PF, present-focus; Awr, awareness; Acc, acceptance; Tot, total score.

### Biomarkers and genomics

The allostatic load index did not show any significant between-group effects among the three treatment arms; post-intervention allostatic load index score changes of the yoga treatment arm showed difference with the control arm (raw *p* = 0.017), but it did not survive correction for multiple comparisons. The allostatic load index showed slight elevation at the follow-up timepoint within the control arm (raw *p* = 0.048), but it did not survive correction for multiple comparisons; there were no indications of change within the meditation or yoga treatment arm at any of the study timepoints (Table 3). No significant within-group or between-group effects were found from genomic analyses (Table 4).

**Table 3:** Changes of allostatic load index components from baseline (week 0) to immediate end of treatment (week 9), and from baseline (week 0) to follow-up (week 26).

	Baseline			Post-intervention-baseline			Follow-up-baseline		
	Est	SE	*p*	Est	SE	*p*	Est	SE	*p*
Overall									
SBP (mm Hg)	112.5	0.84	-	−0.69	0.75	0.36	−0.84	0.79	0.29
DBP (mm Hg)	71.62	0.56	-	−1.14	0.52	0.03	−1.16	0.57	0.04
Pulse Rate (BPM)	70.15	0.83	-	1.14	0.88	0.20	−1.06	0.75	0.16
BMI (kg/m^2^)	25.20	0.31	-	0.01	0.05	0.86	0.11	0.08	0.21
Waist/hip Ratio	0.79	0.004	-	0.002	0.003	0.57	0.001	0.003	0.74
Chol (mg/dL)	172.1	-	-	1.01	-	0.23	1.01	-	0.21
HDL (mg/dL)	58.94	-	-	1.01	-	0.41	1.01	-	0.40
HbA1c (%)	5.43	-	-	1.00	-	0.69	1.00	-	0.85
CRP (mg/dl)	0.80	-	-	0.98	-	0.80	1.15	-	0.13
Urinary Cortisol (ug/12-h)	1.40	-	-	1.00	-	0.99	0.96	-	0.51
Urinary Epi (ug/12-h)	1.81	-	-	0.97	-	0.61	0.82	-	<0.001
Urinary Norepi (ug/12-h)	16.82	-	-	0.92	-	0.12	0.89	-	0.036
DHEA-S (ug/dL)	147.8	-	-	0.97	-	0.08	0.97	-	0.056
IL-6 (pg/ml)	0.39	-	-	1.78	-	<0.001	1.77	-	<0.001
Insulin (uIU/mL)	5.19	-	-	1.04	-	0.31	1.02	-	0.66
ALI (0–15)	3.14	-	-	1.03	-	0.59	1.09	-	0.05
Stress education									
SBP (mm Hg)	112.5	0.84	-	0.45	1.18	0.70	−2.32	1.28	0.07
DBP (mm Hg)	71.62	0.56	-	−1.07	0.83	0.20	−2.39	0.90	0.009
Pulse Rate (BPM)	70.15	0.83	-	2.17	1.38	0.12	−0.92	1.14	0.42
BMI (kg/m^2^)	25.20	0.31	-	−0.03	0.08	0.71	0.10	0.14	0.49
Waist/hip Ratio	0.79	0.004	-	0.007	0.004	0.10	−0.000	0.005	0.99
Chol (mg/dL)	172.1	-	-	1.01	-	0.62	1.00	-	0.91
HDL (mg/dL)	58.94	-	-	0.10	-	0.89	0.98	-	0.41
HbA1c (%)	5.43	-	-	1.01	-	0.18	1.00	-	0.93
CRP (mg/dl)	0.80	-	-	0.93	-	0.58	1.09	-	0.57
Urinary Cortisol (ug/12-h)	1.40	-	-	1.11	-	0.22	1.02	-	0.87
Urinary Epi (ug/12-h)	1.81	-	-	1.03	-	0.71	0.84	-	0.046
Urinary Norepi (ug/12-h)	16.82	-	-	1.01	-	0.91	0.92	-	0.31
DHEA-S (ug/dL)	147.8	-	-	0.94	-	0.09	0.94	-	0.032
IL-6 (pg/ml)	0.39	-	-	2.50	-	<0.001	1.37	-	0.17
Insulin (uIU/mL)	5.19	-	-	1.08	-	0.21	1.02	-	0.75
ALI (0–15)	3.14	-	-	1.13	-	0.12	1.15	-	0.048
Meditation									
SBP (mm Hg)	112.5	0.84	-	−0.74	1.24	0.55	0.31	1.37	0.82
DBP (mm Hg)	71.62	0.56	-	−0.92	0.87	0.29	−0.01	0.95	0.99
Pulse Rate (BPM)	70.15	0.83	-	1.11	1.45	0.45	−0.25	1.21	0.83
BMI (kg/m^2^)	25.20	0.31	-	0.05	0.08	0.58	0.02	0.15	0.88
Waist/hip Ratio	0.79	0.004	-	−0.001	0.004	0.84	−0.001	0.005	0.86
Chol (mg/dL)	172.1	-	-	1.03	-	0.03	1.00	-	0.87
HDL (mg/dL)	58.94	-	-	1.00	-	0.78	0.98	-	0.29
HbA1c (%)	5.43	-	-	1.00	-	0.74	1.00	-	0.77
CRP (mg/dl)	0.80	-	-	1.06	-	0.67	1.14	-	0.40
Urinary Cortisol (ug/12-h)	1.40	-	-	0.96	-	0.63	0.99	-	0.89
Urinary Epi (ug/12-h)	1.81	-	-	0.95	-	0.51	0.78	-	0.011
Urinary Norepi (ug/12-h)	16.82	-	-	0.88	-	0.13	0.89	-	0.18
DHEA-S (ug/dL)	147.8	-	-	0.95	-	0.16	1.00	-	0.88
IL-6 (pg/ml)	0.39	-	-	1.53	-	0.05	2.41	-	<0.001
Insulin (uIU/mL)	5.19	-	-	1.10	-	0.11	0.99	-	0.87
ALI (0–15)	3.14	-	-	1.12	-	0.18	1.11	-	0.17
Yoga									
SBP (mm Hg)	112.5	0.84	-	−1.79	1.28	0.17	−0.50	1.37	0.71
DBP (mm Hg)	71.62	0.56	-	−1.43	0.90	0.11	−1.07	0.96	0.26
Pulse Rate (BPM)	70.15	0.83	-	0.14	1.50	0.92	−1.99	1.21	0.10
BMI (kg/m^2^)	25.20	0.31	-	0.01	0.09	0.93	0.20	0.15	0.19
Waist/hip Ratio	0.79	0.004	-	−0.002	0.005	0.71	0.004	0.005	0.45
Chol (mg/dL)	172.1	-	-	0.99	-	0.58	1.03	-	0.06
HDL (mg/dL)	58.94	-	-	1.02	-	0.20	1.08	-	0.001
HbA1c (%)	5.43	-	-	1.00	-	0.80	1.00	-	0.90
CRP (mg/dl)	0.80	-	-	0.95	-	0.73	1.24	-	0.14
Urinary Cortisol (ug/12-h)	1.40	-	-	0.94	-	0.50	0.89	-	0.25
Urinary Epi (ug/12-h)	1.81	-	-	0.95	-	0.53	0.85	-	0.08
Urinary Norepi (ug/12-h)	16.82	-	-	0.88	-	0.12	0.86	-	0.08
DHEA-S (ug/dL)	147.8	-	-	1.00	-	0.99	0.96	-	0.25
IL-6 (pg/ml)	0.39	-	-	1.48	-	0.08	1.68	-	0.037
Insulin (uIU/mL)	5.19	-	-	0.95	-	0.37	1.05	-	0.50
ALI (0–15)	3.14	-	-	0.86	-	0.08	1.03	-	0.75

Uncorrected raw *p*-values are reported in the table, those with *p* ≤ 0.001 survived correction for multiple comparisons with corrected *p* < 0.05. Est, estimated means (i.e., estimates at baseline are the same across group interventions given that this is a shared-baseline analysis); SE, standard error; SBP, systolic blood pressure; DBP, diastolic blood pressure; BMI, body mass index; Chol, total cholesterol; HDL, high-density lipoprotein; HbA1c, hemoglobin A1c; CRP, C-reactive protein; Epi, epinephrine; Norepi, norepinephrine; DHEA-S, serum dehydroepiandrosterone sulfate; IL-6, serum interleukin-6; Insulin, serum insulin; ALI, allostatic index.

**Table 4:** Changes in genomic regulation from baseline (week 0) to immediate end of treatment (week 9), and from baseline (week 0) to follow-up (week 26).

	Baseline			Post-intervention vs. baseline			Follow-up vs. baseline		
	Est	SE	*p*	Est	SE	*p*	Est	SE	*p*
Overall									
NF-κB Path	6,185	21.67	-	−16.25	27.02	0.55	10.50	30.17	0.73
NF-κB-Pro	6,063	27.13	-	−37.90	36.67	0.30	−3.23	38.80	0.93
NF-κB-Anti	7,704	12.21	-	6.07	12.39	0.62	2.72	14.27	0.85
TNFR2	5,906	30.30	-	−20.41	33.89	0.55	25.06	39.51	0.53
NTHi	5,726	33.48	-	5.53	42.76	0.90	26.19	45.84	0.57
Inflam Resp	2,632	13.33	-	27.99	15.18	0.07	28.87	16.53	0.08
Oxi Phos	6,034	32.24	-	−46.38	43.67	0.29	−24.06	43.17	0.58
IL-6 JAK STAT3	3,658	16.39	-	23.44	18.74	0.21	46.22	21.88	0.04
Stress education									
NF-κB Path	6,185	21.67	-	−32.26	39.94	0.42	1.41	44.21	0.98
NF-κB-Pro	6,063	27.13	-	−67.50	52.90	0.20	−30.43	56.66	0.59
NF-κB-Anti	7,704	12.21	-	−1.83	19.37	0.92	40.06	22.03	0.07
TNFR2	5,906	30.30	-	−66.36	54.18	0.21	33.26	60.52	0.58
NTHi	5,726	33.48	-	−48.95	60.91	0.42	33.75	67.60	0.62
Inflam Resp	2,632	13.33	-	7.88	22.88	0.73	25.42	25.33	0.73
Oxi Phos	6,034	32.24	-	−80.16	66.06	0.23	−6.94	66.50	0.92
IL-6 JAK STAT3	3,658	16.39	-	20.24	28.37	0.48	59.74	32.78	0.07
Meditation									
NF-κB Path	6,185	21.67	-	2.88	42.04	0.95	22.47	48.56	0.64
NF-κB-Pro	6,063	27.13	-	−8.61	55.54	0.88	−17.98	62.17	0.77
NF-κB-Anti	7,704	12.21	-	33.48	20.49	0.10	0.52	24.39	0.98
TNFR2	5,906	30.30	-	39.14	56.26	0.49	43.48	66.95	0.52
NTHi	5,726	33.48	-	92.53	63.87	0.15	25.90	74.47	0.73
Inflam Resp	2,632	13.33	-	22.54	24.12	0.35	30.05	28.08	0.29
Oxi Phos	6,034	32.24	-	30.95	69.64	0.66	−62.02	73.65	0.40
IL-6 JAK STAT3	3,658	16.39	-	8.51	29.92	0.78	45.02	36.18	0.22
Yoga									
NF-κB Path	6,185	21.67	-	−19.35	42.83	0.65	7.63	46.62	0.87
NF-κB-Pro	6,063	27.13	-	−37.59	56.57	0.51	38.70	59.73	0.52
NF-κB-Anti	7,704	12.21	-	−13.43	20.87	0.52	−32.42	23.27	0.17
TNFR2	5,906	30.30	-	−34.01	57.34	0.55	−1.57	63.97	0.98
NTHi	5,726	33.48	-	−26.99	65.08	0.68	18.92	71.24	0.79
Inflam Resp	2,632	13.33	-	53.55	24.60	0.031	31.14	26.73	0.25
Oxi Phos	6,034	32.24	-	−89.93	71.06	0.21	−3.23	70.25	0.96
IL-6 JAK STAT3	3,658	16.39	-	41.56	30.49	0.17	33.91	34.60	0.33

Uncorrected raw *p*-values are reported in the table, none of the results survived correction for multiple comparisons. Est, estimated means (i.e., estimates at baseline are the same across group interventions given that this is a shared-baseline analysis); SE, standard error; NF-κB Path, Biocarta NF-κB pathway; NF-κB Pro, Biocarta NF-κB pathway pro-inflammatory; NF-κB Anti, Biocarta NF-κB pathway anti-inflammatory; TNFR2, tumor necrosis factor receptor 2; NTHi, Nontypeable *Haemophilus influenzae*; Inflam Resp, Hallmark inflammatory response; Oxi Phos, Hallmark oxidative phosphorylation; IL-6 Jak STAT3, Hallmark IL-6 JAK STAT3 signaling.

Results on individual components of the allostatic load were also analyzed within each group, and most of them showed no significant changes (Table 3). Directional increase with IL-6 were observed within the stress education arm (raw *p* < 0.001, corrected *p* < 0.05) at the post-intervention timepoint, within the meditation arm at the post-intervention (raw *p* = 0.05, did not survive correction) and follow-up (raw *p* < 0.001, corrected *p* < 0.05) time points, and within the yoga arm at the follow-up time point (raw *p* = 0.037, did not survive correction). The yoga treatment arm also showed elevated HDL at the follow-up timepoint (raw *p* = 0.001, corrected *p* < 0.05).

### Correlations between changes in psychosocial measures and biomarkers

Some correlations were identified between post-intervention changes of psychological measures and biomarkers, however, none of them survived correction for multiple comparisons.

Pooling together all research participants from all three arms, post-intervention changes of PSS-10 scores were positively correlated with changes of allostatic load index (*r* = 0.22, raw *p* = 0.010). This correlation was only apparent in the stress education arm (*r* = 0.36, raw *p* = 0.013) but not in the meditation or yoga arm. None of the above statistics survived correction for multiple comparisons.

Also with pooled data, post-intervention decreases in PSS-10 scores were associated with post-intervention decreases in both diastolic blood pressure (*r* = 0.16, raw *p* = 0.04) and systolic blood pressure (*r* = 0.20, raw *p* = 0.013). Within the meditation group, decreases of PSS-10 scores were associated with decreases of blood pressure (systolic blood pressure: *r* = 0.35, raw *p* = 0.011; diastolic blood pressure: *r* = 0.46, raw *p* < 0.001). Within the yoga group, decreases of PSS-10 scores correlated with increases of serum insulin levels (*r* = −0.31, raw *p* = 0.034). None of the above statistics survived correction for multiple comparisons.

In pooled data with all participants, post-intervention score changes of PWBS-SA showed a negative correlation with systolic blood pressure (*r* = −0.19, raw *p* = 0.015), allostatic load index (*r* = −0.22, raw *p* = 0.009), and with HDL (*r* = −0.24, raw *p* = 0.003), while the CAMS-R Awareness subscale showed a negative correlation with systolic blood pressure (*r* = −0.22, raw *p* = 0.007). Within the yoga arm, post-intervention changes of c-reactive protein (CRP) were negatively correlated with CAMS-R-Awr (*r* = −0.41, raw *p* = 0.008), and post-intervention changes of serum insulin were negatively correlated with the PANAS-NA (*r* = −0.37, raw *p* = 0.009). Within the meditation arm, post-intervention changes of diastolic blood pressure were positively correlated with STAI-SS (*r* = 0.31, raw *p =* 0.029). None of the above statistics survived correction for multiple comparisons.

## Discussion

This 3-arm RCT compared meditation, yoga, and an active control among chronically stressed but otherwise healthy adults with a large battery of outcome measures. The three arms did not show significant differences in any of the outcome measures. Most psychological measures demonstrated significant post-intervention improvements within all study arms, but physiological measures did not show significant improvements within any study arms. Several psychological questionnaires showed strong correlations with physiological measures, such as PSS-10, PWBS, CAMS-R and STAI-SS, but did not survive multiple comparison.

Self-report measures are often criticized for subjectivity, and there have been reports of biological differences in the absence of psychological differences between mind–body interventions and controls, and vice versa (Joss et al., 2024a; Joss et al., 2024b). However, there are also limitations with physiological measures because they can be affected by diurnal fluctuations and many life style factors such as food and smoking (Garde et al., 2009). For example, prior research suggests cortisol response in laboratory-induced acute stress can more accurately reflect intervention effects than accumulative cortisol (Rogerson et al., 2024). Because all the physiological measures used in this study were global accumulative measures, which could be influenced by many factors in real life, the allostatic measures used in this study may not be sensitive enough to detect intervention-related changes. Prior studies on the effects of mind–body interventions on gene expression usually utilized a hypothesis-free gene set enrichment analysis (GESA) approach (Bhasin et al., 2018; Bhasin et al., 2013; Kuo et al., 2015), whereas this study focused on specific gene sets such as the BioCarta NF-kB Pathway based on our prior research (Bhasin et al., 2018; Bhasin et al., 2013; Dusek et al., 2008a; Dusek et al., 2008b; Kuo et al., 2015). It is possible that this hypothesis-driven approach might have been too restricted in its ability to detect potentially important changes in genetic expression related to mind–body interventions. Therefore, the mostly null findings with physiological outcome measures in this study could have been related to the research methodology of measuring global accumulative biomarkers and using a hypothesis-driven genomic analysis approach.

Multiple review articles have demonstrated overall mixed findings regarding the effects of mind–body programs on stress biomarkers such as cortisol (O'Leary et al., 2016) and IL-6 (Bower and Irwin, 2016; Moreno, 2024), despite some reports of post-intervention reduction (Walsh et al., 2016). For example, one recent study reported post-intervention elevation of IL-6 among older adults (Lindsay et al., 2022), which was interpreted as indicative of adaptive boost of immune competence for fighting off infections among older adults (Lindsay et al., 2022; Moreno, 2024). A recent meta-analysis of 105 meditation studies with total *N* = 3,826 found overall small effect size on immune functioning including IL-6 levels (Oyler et al., 2023). Although our study showed increased IL-6 within the stress education and meditation treatment arms, the elevated values were still within the normal range for healthy adults (Said et al., 2021). The physiological or biopsychosocial mechanisms for IL-6 level fluctuations within the normal range among healthy adults are still not well understood (Picotte et al., 2009). Our study also found elevated HDL in the yoga treatment arm at the follow-up timepoint, which is consistent with findings on the association of yoga and HDL in a recent meta-analysis of 53 studies with a total sample size of *N =* 13,191 (Ghazvineh et al., 2022). Our findings add to the literature of mixed findings on the effects of mind–body interventions for stress biomarkers for consideration in future research.

A major limitation of this study is the use of a relatively healthy study sample. This study only enrolled chronically stressed but otherwise healthy adults with no mental illness. Any prospective participants with DSM axis-I disorders were excluded. The purpose of choosing the healthy adult study population was to investigate the effects of mind–body interventions while avoiding confounding factors associated with any particular medical or mental health disorders. This kind of healthy adult sample is representative of a large number of healthy adult participants of mind–body programs in the general public, therefore this study design has generalizability for the public. Nevertheless, in this study, the healthy adult sample might have led to floor effects that limited the detectability of intervention-related physiological changes with existing technology within the short timeframe of the study, whereas study populations with psychopathology could have larger effects that are more detectable (Wielgosz et al., 2019). For example, a recent systematic review of 44 meta-analyses of mindfulness-based interventions (k = 336 RCTs, *N* = 30,483 participants) suggests strongest effects were observed from disease populations, whereas findings from healthy adult populations were inconsistent with overall small effect sizes when compared with active control conditions (Goldberg et al., 2022).

As discussed above, the stringent eligibility criteria of this study excluded any prospective participants that met diagnosis criteria for any DSM axis-I disorders; future studies may consider loosening the eligibility criteria to include patients with mild or moderate levels of Major Depressive Disorder and Generalized Anxiety Disorder, which also represent a large number of participants of mind–body programs in the general public. Prospective participants on any psychiatric medications were excluded from this study out of concerns of affecting genomic measures, but such exclusion criteria may not be necessary for future clinical trials focused on testing the clinical efficacy of mind–body programs. The “chronically stressed” criterion was based on self-report, which could have susceptibility to inaccuracies and biases, because self-reported perceived stress is not necessarily equivalent to physiological stress levels, for example, someone could be experiencing chronic stress but lacks the awareness to realize they have been stressed thus under-report the “perceived stress” levels, whereas someone could be hyper-sensitive to the fluctuation in stress levels without actually having high levels of stress biomarkers. Nevertheless, although biomarker-based eligibility criteria might have been more objective, there is currently no consensus on appropriate stress biomarkers with feasible clinical application, and it is time consuming and resource demanding to utilize the composite allostatic load index with 14 biomarkers for screening purposes.

Furthermore, the stress education active control arm (Hoge et al., 2013) might have had unexpectedly strong stress reduction elements, such as using audio books as daily home practice and raising awareness on diet and exercise. Such an active control condition has shown similar effects in reducing stress, psychological symptoms and blood pressure compared to mind–body interventions (Dusek et al., 2008a; Dusek et al., 2008b; Joss et al., 2024a; Joss et al., 2024b; Joss et al., 2024c). Most importantly, recent meta-analyses have demonstrated that mind–body interventions often had no superiority over active control conditions in prior studies (Goldberg et al., 2022), therefore the lack of between-group effects in this study is consistent with the existing literature.

There are several other limitations with this study. (1) Given the above-mentioned limitations with the active control arm, this study could have benefited from including a passive control arm, such as a waitlist or “do nothing” control arm, which might have been able to provide information on longitudinal psychological and physiological changes without any interventions. (2) The null findings in this study could have been related to the limitations with the instruments and assays used for screening and assessments. Newer technologies, such as AI-based facial biomarkers (Al-Jebrni et al., 2020; Wang et al., 2025), is more objective than self-report measures and potentially more specific and sensitive than traditional physiological measures. Therefore, future studies should consider utilizing these AI based technologies. (3) Lack of study sample demographic diversity is another limitation, which compromised the generalizability of study findings. Despite all the limitations, this study can still provide methodological insights for future mind–body research.

## Data Availability

The raw data supporting the conclusions of this article will be made available by the authors, without undue reservation.
