# Supplementary material for: Psychological assessments, allostatic load and gene expression analyses in a randomized controlled trial comparing meditation, yoga, and stress education
Source: Front Psychol. 2025 Sep 12;16:1653242. doi: 10.3389/fpsyg.2025.1653242 (PMC12463968; doi:10.3389/fpsyg.2025.1653242)
Supplement: Supplementary file 1 [file Data_Sheet_1.pdf]

Supplementary material for

Psychological Assessments, Allostatic Load and Gene Expression Analyses in a Randomized Controlled Trial Comparing Meditation, Yoga, and Stress Education

This supplementary material contains public information about the research grant that supported this study, awarded by the National Center for Complementary and Integrative Health (NCCIH) of the National Institute of Health of the United States of America (grant # 5R01AT006464). The grant information can be assessed at the URL below:
https://reporter.nih.gov/project-details/8541701

Grant Title:
Quantification of Outcome Measures for Mind Body Interventions

Grant Abstract:
Research on mind-body interventions has established their capability for reducing perceived chronic psychological stress and its symptoms and enhancing well-being and wellness through induction of the relaxation response (RR), a coordinated, systemic psychophysiological and behavioral response that is opposite to the well-known fight-or-flight/stress response. However, there is little known about the characteristics of previously used outcome measures for stress reduction including their precision, reliability, and validity, the importance of collection conditions, the relationship between objective and subjective measures and the behavior of these outcome measures with different mind-body interventions. This application aims to address these issues with a randomized controlled trial of an 8-week intervention with three treatment arms of meditation, yoga and a stress-education behavioral placebo control in normal, healthy subjects with moderate to severe perceived stress. A comprehensive battery of: 1) subjective self-report measures of perceived psychological stress and mental health and well-being; 2) genomic expression measures from blood samples; and 3) biochemical assays from blood, saliva and urine samples will be acquired at baseline, end- treatment and long-term follow-up. All outcome measures will be subjected to comprehensive comparative statistical analyses to determine their relative precision, reliability and validity and the strength of correlations between them for all three treatment conditions. This study will provide novel and important information regarding the most reliable, accurate and cost-effective outcome measures and the optimum method of their administration in mind-body medicine research protocols. The results of this study will also provide new and valuable information on the role and potential efficacy of the relatively new genomic expression outcome measure and its relationship to previously used outcome measures in mind-body medicine. Finally, this study will determine how consistent all of these measures are between a purely cognitive RR-based mind-body intervention (meditation) and another that

includes a physical component (yoga) and whether there are subtle differences between them in these outcome measures.

Public Health Relevance Statement:
This study aims to provide novel information on the relative precision, reliability and validity of psychological self-report, biochemical and genomic outcome measures for mind body interventions, and on the strength of correlations between them. This information will be important for the determination of the most reliable, accurate and cost-effective outcome measures and the optimum method of their administration in future mind body medicine research protocols. This will therefore serve to strengthen the quality and validity of future research studies of mind body interventions, which are both efficacious and cost-effective, for both prevention and treatment of medical and psychological conditions.
